# Altered HDL Phospholipid and Fatty Acid Profile in MASLD: A Possible Explanation for the Increased CVD Risk

**DOI:** 10.3390/ijms26136148

**Published:** 2025-06-26

**Authors:** Sofia Kartsoli, Christina E. Kostara, Athanasios Papathanasiou, Vasilis Tsimihodimos, Eleni T. Bairaktari, Dimitrios K. Christodoulou

**Affiliations:** 1 Department of Gastroenterology, School of Health Sciences, Faculty of Medicine, University of Ioannina, 45110 Ioannina, Greece; kartsolisofia@gmail.com; 2 Laboratory of Clinical Chemistry, School of Health Sciences, Faculty of Medicine, University of Ioannina, 45110 Ioannina, Greece; chkostara@uoi.gr (C.E.K.); ebairakt@uoi.gr (E.T.B.); 3 Department of Internal Medicine, School of Health Sciences, Faculty of Medicine, University of Ioannina, 45110 Ioannina, Greece; a.papathanasi3@gmail.com (A.P.); tsimiho@gmail.com (V.T.)

**Keywords:** metabolic dysfunction-associated steatotic liver disease, coronary artery disease, CVD risk, HDL, lipoproteins, NMR, lipidomics, phospholipids, sphingolipids, fatty acids

## Abstract

Metabolic dysfunction-associated steatotic liver disease (MASLD) has been consistently linked to increased risk of cardiovascular disease (CVD). HDL lipoproteins may serve as a possible link in this association through their hepatic synthesis and atheroprotective properties. Serum samples were collected from 51 MASLD patients (diagnosed by abdominal ultrasound), 40 with coronary artery disease, and 50 healthy controls. HDL lipid profiles were investigated by proton nuclear magnetic resonance (^1^H NMR) spectroscopy. Patients with MASLD exhibit an increased percentage of lysophosphatidylcholine and sphingolipid content, mainly due to increased ceramides, and a reduced percentage of phosphatidylcholine, phosphatidylethanolamine, and phosphatidylinositol compared to controls. The % content of total and individual polyunsaturated fatty acids including linoleic, docosahexaenoic, eicosapentaenoic, and arachidonic acid was found to be reduced in patients with MASLD, while saturated fatty acid content was increased compared to the control group. These alterations in fatty acid composition were observed also in CAD patients compared to controls but were more pronounced in CAD patients. Compared to CAD patients, those with MASLD showed an increased content of sphingolipids, ceramides, and glycerolipids and a reduced content of phosphatidylinositol. Changes observed in the lipid composition of HDL lipoproteins in MASLD patients may impair the protective properties of HDL particles, contributing to increased CVD risk.

## 1. Introduction

Metabolic dysfunction-associated steatotic liver disease (MASLD), the most common chronic liver disorder worldwide, is becoming rapidly a global health problem. The worldwide prevalence of MASLD is estimated to be 38% and in 2040 is predicted to rise up to 55% [1]. MASLD is characterized by lipid accumulation in the liver in the presence of at least one cardiometabolic risk factor and encompasses a wide spectrum of hepatic pathologies, ranging from simple steatosis to metabolic dysfunction-associated steatohepatitis (MASH) and then finally to cirrhosis and hepatocellular carcinoma [2]. Although the mechanisms governing the progression from hepatic steatosis to MASH remain poorly understood, alterations in hepatic lipid metabolism and lipotoxicity are considered major drivers of the development of MASH [3].

The disease strongly associates with obesity, insulin resistance, and dyslipidemia, the main components of metabolic syndrome, and precedes the development of this condition [2]. Notably, all the above serve also as cardiovascular (CVD) risk factors, and individuals with MASLD have a greater risk for developing fatal and non-fatal cardiovascular events [4]. Current cardiovascular prevention strategies focus on managing modifiable risk factors across populations, including early identification and treatment of dyslipidemia and metabolic dysfunction, as outlined by recent consensus recommendations [5]. Whether MASLD contributes actively to the pathogenesis of CVD or represents an innocent bystander has not yet been clarified. The liver is the key site of serum lipoprotein uptake and secretion, and liver steatosis is associated with atherogenic serum lipid changes, such as decreased high-density lipoprotein (HDL) levels, as well as elevated plasma triglycerides (TGs) and low-density lipoprotein (LDL) levels [2].

The atheroprotective effects of HDL and the inverse correlation between high-density lipoprotein cholesterol (HDL-C) levels and the risk of coronary artery disease (CAD) are well known [6]. High-density lipoprotein mediates reverse cholesterol transport and possesses anti-inflammatory, antioxidant, anti-coagulant, and pro-fibrinolytic properties [6]. Of note, a growing body of evidence suggests that the lipid and protein composition of HDL, rather than HDL cholesterol levels, capture better the HDL functionality and cardiovascular protection [7]. These lipoprotein particles are subjected to several structural changes, which compromise their properties and their subsequent biological activities [8]. In fact, chronic inflammation has been linked with reduced phospholipid content of HDL particles and changes in modulating enzymes resulting in impaired HDL anti-atherogenic functions [9]. Currently, only one study has investigated the fatty acid composition of HDL particles in patients with MASLD [10], while no studies have examined the whole lipid composition of HDL lipoproteins in these patients.

Lipidomics, a subset of metabolomics, is a new rapidly growing research field and represents the detailed characterization of lipid molecular species (lipidome) and their biological role in a given matrix, including cells, tissue, and biological fluid [11]. The identification and characterization, as well as the quantification, of lipid molecular species in a biological matrix, through lipidomics, serve as a powerful approach to obtaining a comprehensive overview of whole-lipid metabolism in a biological system or even in a specific disease state [12]. Disturbances in the lipidome can be interrogated using platforms consisting of high-field-resolution nuclear magnetic resonance (NMR) spectroscopy coupled with multivariate statistical methods. NMR spectroscopy is a powerful and reliable tool to provide structural elucidation and qualitative and quantitative analyses of lipid molecules in biological samples with high reproducibility [13]. The analysis of the complex lipidomic datasets generated by multivariate statistical analysis (MVA) represents a powerful exploratory tool for identifying causal links between biomarkers and disease phenotypes.

In the present study, we used an NMR-based lipidomic approach to investigate the alterations occurring in the lipid composition of HDL particles in patients with MASLD and compare them to those in patients with coronary artery disease. Analyzing the lipidome of HDL lipoproteins in MASLD may provide insights into the structural changes in these particles and identify compositional alterations of each lipid class that contribute to increased CVD risk in these patients. 

## 2. Results

### 2.1. Characteristics of Study Population

The main demographic data and serum lipid parameters of the three populations are presented in Table 1. The three groups were well matched for age, gender, and serum lipid profile (triglycerides; total, HDL, LDL, and non-HDL cholesterol) to minimize their confounding effect on the data analysis.

### 2.2. HDL Lipidome: MASLD vs. Control Group

To demonstrate the impact of MASLD on the HDL lipidome, we performed both a targeted and untargeted lipidomic approach. For these approaches, lipid components were quantified from selected well-resolved signals in the NMR fingerprint (Figure S1, Table S1 in the Supplementary Data).

#### 2.2.1. Targeted Lipid Analysis of HDLs

As described below, the targeted lipidomic analysis revealed that MASLD was associated with qualitative and quantitative alterations in HDLs, mainly in free cholesterol and individual phospholipids molecules, as well as in fatty acids, with which they are esterified.

Specifically, between patients with MASLD and controls, the percentages of total and esterified cholesterol (EC), triglycerides, total phospholipids (PLs), total core (triglycerides and esterified cholesterol), and surface (phospholipids and free cholesterol) lipids, as well as the ratio of TG/EC, were not significantly different (Table 2). Free cholesterol (FC) content was significantly higher in MASLD patients compared to controls, resulting in a higher ratio of FC/PLs, whereas the ratios of EC/FC and EC/Total Cholesterol were significantly lower (Table 2).

Table 3 shows the alterations that occurred in the percentages of total glycerophospholipids (GPLs), total ether glycerolipids (ether GLs), and total sphingolipids (SLs), as well as in individual phospholipid molecules that were quantified from characteristic well-resolved signals in the proton NMR fingerprint. The content of total GPLs was significantly lower in MASLD patients compared to controls, whereas that of total SLs was significantly higher. Ether GLs content was higher in the MASLD group compared to controls but without significance.

As shown in Table 3, the pattern of individual GPLs in MASLD was different from that in control individuals. Specifically, the percentages of phosphatidylcholine (PC), phosphatidylethanolamine (PE), and phosphatidylinositol (PI) were significantly lower in patients with MASLD, whereas lysophosphatidylcholine (LPC) content was significantly higher (Table 3). The percentages of total ether GLs and plasmalogens were similar between the two groups, while that of the rest of the ether GLs, mainly attributed to the platelet-activating factor (PAF), was significantly higher in MASLD compared to controls (Table 3). Finally, total SLs content was higher in MASLD compared to controls, mainly due to a significant increase in the rest of the SLs attributed to ceramide and sphingosine-1-phosphate (S1P). The percentage of sphingomyelin (SM), the second most abundant phospholipid on the particle’s surface, was higher in MASLD compared to controls but without significance. The above-described alterations resulted in changes in the molecular ratios PC/SM and PC/LPC, while the SM/LPC ratio remained unchanged.

The fatty acid composition of esterified lipids carried by HDLs is shown in Table 4. Patients with MASLD presented with a significantly higher percentage of saturated fatty acids (SFAs) and a lower percentage of unsaturated fatty acids (UFAs) compared to the control group. It is worth mentioning that this decrease is mainly due to polyunsaturated fatty acids (PUFAs) with a significance of <0.01, whereas the monounsaturated fatty acids (MUFAs) content was higher but without significance (Table 4). The percentages of individual PUFA, including linoleic acid (LA); the sum of eicosapentaenoic and arachidonic acid (EPA + AA); and docosahexaenoic acid (DHA) were significantly lower in MASLD compared to controls. The above-described changes in the fatty acid pattern resulted in significantly higher ratios of SFA to UFA and of SFA to PUFA in MASLD compared to controls.

#### 2.2.2. Untargeted Lipid Analysis of HDLs

**Figure 1:**
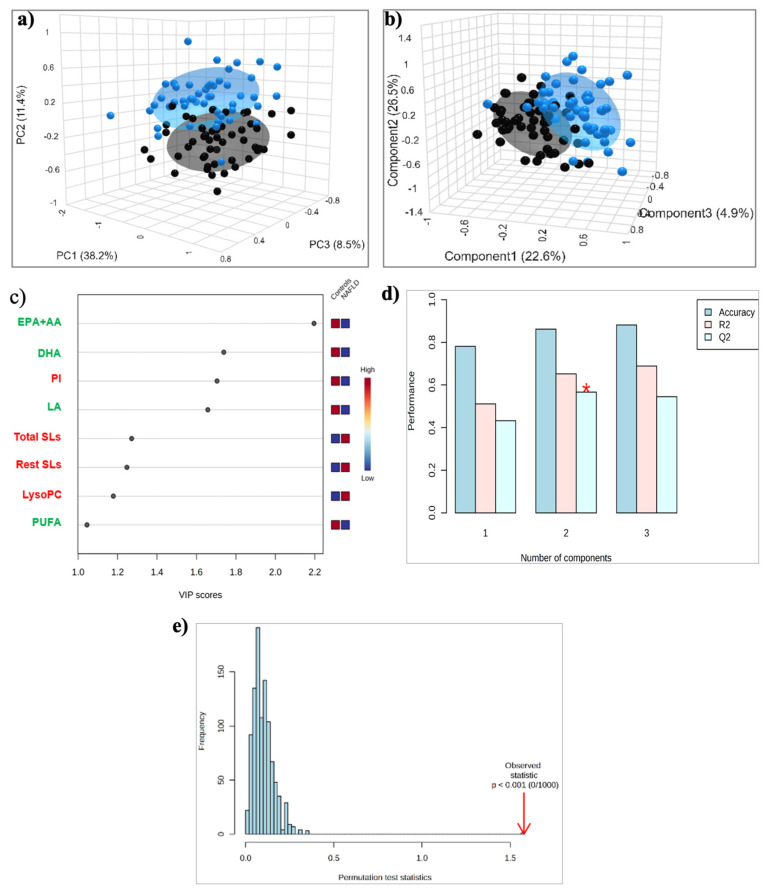
(**a**) PCA and (**b**) PLS-DA score plots for the 50 Controls (black circles) and 51 patients with MASLD (blue circles); (**c**) The top 8 most discriminating HDL lipids discriminating MASLD from Controls, ranked by variable importance in projection (VIP) scores of PLS-DA model. VIP scores ≥ 1 were considered significant; (**d**) Cross-validation; (**e**) Permutation test. *: *p* < 0.05. Key: DHA, Docosahexaenoic acid; EPA+AA, Eicosapentaenoic and Arachidonic acid; LA, Linoleic acid; LysoPC, Lysophosphatidylcholine; PI, Phosphatidylinositol; PUFA, Polyunsaturated fatty acids; Rest SLs, Rest Sphingolipids; Total SLs, Total Sphingolipids.

Principal component analysis (PCA) was initially performed to examine the natural tendency of grouping or grouping between MASLD patients and control subjects, and to identify potential outliers. The 3D PCA score plot (Figure 1a) showed a clear separation between the two groups, with a minimal area of overlap based on the first principal component, which accounted for 38.2% of the overall variance.

The application of the supervised PLS-DA analysis showed improved discrimination between the two groups, with the samples of both groups tightly clustered together (Figure 1b). A total of eight lipid constituents with variable importance in the projection (VIP) > 1 were identified to contribute significantly to model interpretation (Figure 1c). Total and individual PUFAs, including the sum of EPA + AA, DHA, and LA, as well as PI, exhibited decreased levels, whereas total and the rest of the SLs, and LPC exhibited increased levels in the MASLD group compared to the control (Figure 1c). This separation is estimated by the two quality parameters, R^2^ = 0.652 for the explained variation and Q^2^ = 0.589 for the predictive capability of the resulting model (Figure 1d). In addition, permutation tests (n = 1000 repeats) were performed, and the observed statistical *p*-value (*p* < 0.001) also confirms the validity of the model (Figure 1e).

### 2.3. HDL Lipidome: MASLD vs. CAD Group

Here, we conducted targeted and untargeted analysis in order to investigate whether the changes in the HDL lipidome in MASLD patients are similar to those presented in CAD patients, and thus could contribute or explain the increased CVD risk in these patients.

#### 2.3.1. Targeted Lipid Analysis

Compared to patients with CAD, MASLD patients presented alterations in both surface and core lipids of HDLs, as well as in their fatty acids, indicating that patients with MASLD presented with an atherogenic profile of high CVD risk.

The percentages of total, free, and esterified cholesterol were significantly higher in MASLD patients compared to CAD, whereas that of triglycerides was lower, resulting in a lower TG/EC ratio (Table 2). The ratio of FC/PLs was significantly higher in MASLD patients compared to CAD, whereas the ratios of EC/FC and EC/Total Cholesterol, as well as the content of total phospholipids, core and surface lipids, were similar between the two groups (Table 2).

In addition, MASLD patients presented a significantly lower percentage of total glycerophospholipids compared to CAD patients, mainly due to the lower PI content (Table 3). The percentages of PC and PE were similar between MASLD and CAD patients, and lower compared to those in the control group, whereas those of LPC and the rest of the GPLs were similar between MASLD and CAD patients, and higher compared to those in controls. Compared to CAD, patients with MASLD presented with a higher percentage of ether GLs, mainly due to the rest of the ether GLs, while the plasmalogens content was significantly lower (Table 3). Finally, the content of total SLs, SM, and the rest of the SLs, as well as the ratio of PC/SM, were significantly higher in MASLD patients compared to CAD (Table 3).

Concerning the fatty acid pattern, the percentages of SFA and MUFA were gradually increased from controls to MASLD and then to CAD patients, whereas those of UFA and PUFA (total and individual) were gradually decreased in the same direction. The aforementioned changes resulted in a gradual increase in the ratios of SFA to UFA and of SFA to PUFA.

#### 2.3.2. Untargeted Lipid Analysis 

**Figure 2:**
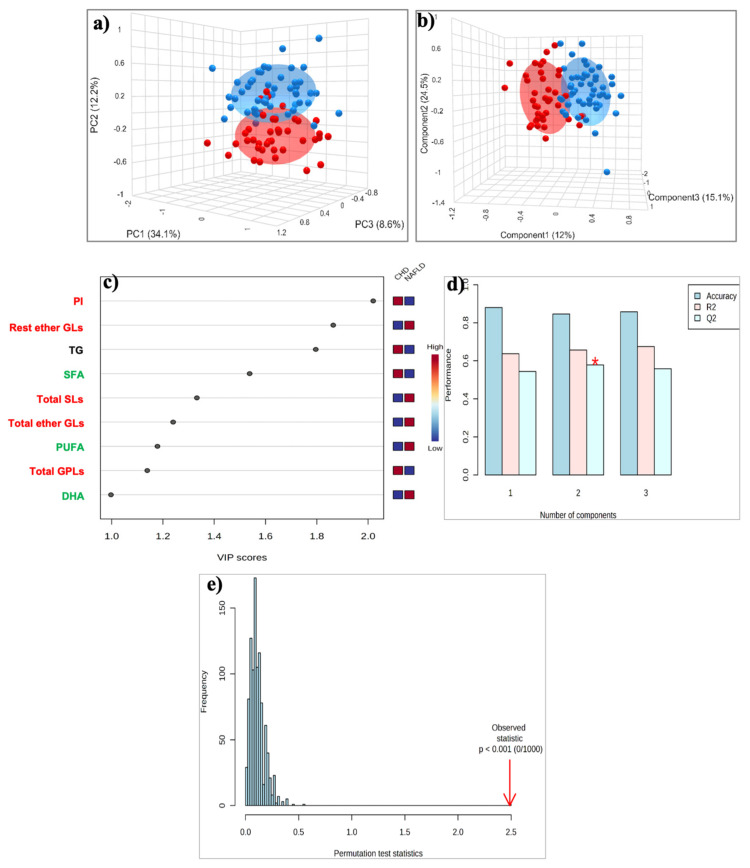
(**a**) PCA and (**b**) PLS-DA score plot for the 51 patients with MASLD (blue circles) and 40 patients with CAD (red circles); (**c**) The top 9 most discriminating HDL lipids discriminating MASLD from Controls, ranked by variable importance in projection (VIP) scores of PLS-DA model. VIP scores ≥ 1 were considered significant; (**d**) Cross-validation, (**e**) Permutation test. *: *p* < 0.05. **Key:** DHA, Docosahexaenoic acid; PI, Phosphatidylinositol; PUFA, Polyunsaturated fatty acids; Rest ether GLs, Rest ether Glycerolipids; SFA, Saturated fatty acids; Total ether GLs, Total ether Glycerolipids; Total GPLs, Total Glycerophospholipids; Total SLs, Total Sphingolipids; TG, Triglycerides.

The 3D PCA score plot (Figure 2a) showed a clear separation between the two groups, with a minimal area of overlap based on the first principal component, which accounted for 34.1% of the overall variance. PLS-DA analysis showed improved discrimination between the two groups, with the samples of both groups to tightly clustered together (Figure 2b). A total of nine lipid constituents with VIP > 1 were identified to contribute significantly to model interpretation (Figure 2c). PI, triglycerides, SFA, and total GPLs exhibited decreased levels, whereas total and the rest of the ether GLs, total SLs, PUFA, and DHA exhibited increased levels in MASLD patients compared to those with CAD (Figure 2c). This separation is estimated by the two quality parameters, R^2^ = 0.657 for the explained variation and Q^2^ = 0.578 for the predictive capability of the resulting model (Figure 2d). Finally, permutation tests (n = 1000 repeats) were performed, and the observed statistical *p*-value (*p* < 0.001) also confirms the validity of the model (Figure 2e).

### 2.4. HDL Lipidome: Control Group vs. MASLD vs. CAD Group

**Figure 3:**
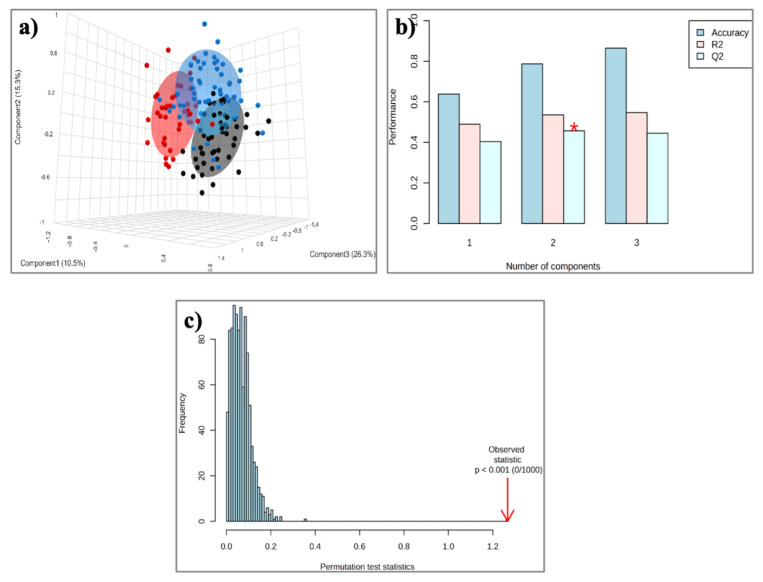
(**a**) PLS-DA score plot for the 50 Controls (black circles), 51 patients with MASLD (blue circles), and 40 patients with CAD (red circles); (**b**) Cross-validation; (**c**) Permutation test. * *p* < 0.0.5.

**Figure 4:**
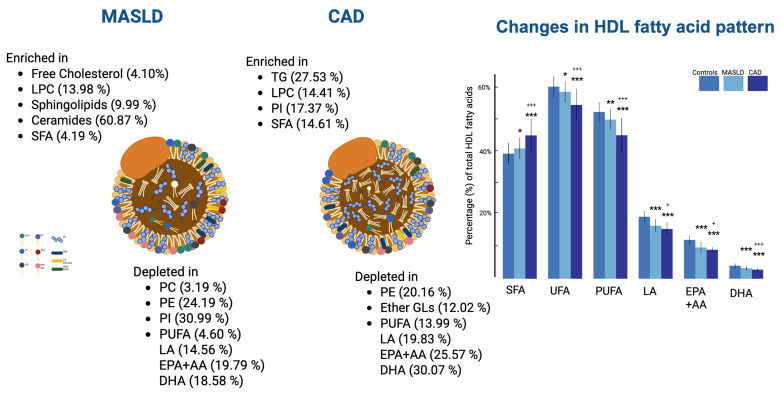
Illustration of major alterations in lipid composition of HDL particles in MASLD and CAD patients compared to control group. Ratios (MASLD/Control and CAD/Control) are shown next to each lipid species. * *p* < 0.05, ** *p* < 0.01, and *** *p* < 0.001 compared to controls; ^+^ *p* < 0.05, ^+++^ *p* < 0.001 compared to patients with MASLD.

The aforementioned qualitative and quantitative alterations in HDL lipids occurring mainly in phospholipids and fatty acids could distinguish gradually the three groups studied (controls, MASLD, and CAD patients), as depicted in the PLS-DA score plot (Figure 3a) created with untargeted analysis, suggesting a relatively high impact of MASLD on the HDL lipid composition. The separation among the three groups is assessed by the following quality parameters of the resulting PLS-DA model: R^2^ = 0.535 and Q^2^ = 0.457 (Figure 3b). Permutation tests (n = 1000 repeats) were performed, and the observed statistical *p*-value (*p* < 0.001) also confirms the validity of the model (Figure 3c). Figure 4 presents an illustration of major alterations in lipid composition of HDL particles in MASLD and CAD patients compared to control group. 

## 3. Discussion

In the present study, we applied a ^1^H-NMR-based lipidomic approach to investigate the lipid composition of HDLs in patients with MASLD compared to healthy individuals and to assess whether these changes induce an atherogenic lipid profile of high cardiovascular risk. The targeted and untargeted lipidomic analysis revealed that the MASLD patients presented significant alterations in the phospholipids and fatty acids pattern of HDLs compared to controls.

In the present study, the lipidomic analysis of HDLs revealed that the phospholipid pattern (glycerophospholipids and sphingolipids) was profoundly altered in MASLD patients. Particularly, the HDL particles’ surface was depleted in glycerophospholipids including PC, PE, and PI, and enriched in LPC in MASLD patients compared to controls. Similar changes have been reported in the liver lipidome of these patients [14,15,16]. One possible explanation is that the liver, as the key site for HDL synthesis and metabolism, directly influences its phospholipid composition [17]. In fact, hepatic ABCA1 is crucial for the formation of nascent HDLs, and the liver actually contributes the majority of the phospholipids in HDL particles [18]. Thus, changes in the HDL composition may partly reflect altered hepatocyte phospholipid content in these patients.

PC is the main glycerophospholipid that is required for the assembly and secretion of lipoproteins. Impaired hepatic PC biosynthesis significantly attenuates the secretion of very-low-density lipoproteins (VLDLs) from the liver and reduces the levels of circulating VLDLs and HDLs [19]. Since the chief route of hepatic lipid secretion is mediated via VLDLs, impaired mobilization of excess liver fat due to reduced PC availability results in liver fat accumulation [19]. Furthermore, decreased hepatic PC content leads to the formation of nascent PC-depleted VLDLs, which are removed more rapidly from the circulation [19], and contributes to hepatic TG accumulation by activating the sterol regulatory element-binding proteins (SREBPs) and subsequently de novo lipogenesis [20]. Thus, decreased VLDL-PC content consequent to impaired PC biosynthesis in the liver could lead to decreased PC content in HDLs. On the other hand, since half of hepatic PC is derived from HDLs, decreased content of HDL-PC may also account for the low hepatic concentration of PC observed in MASLD patients [21]. Considering this, HDL-PC plays a key role in MASLD pathogenesis and is a promising target for studies investigating the metabolic pathways driving disease progression.

The role of PE in MASLD remains uncertain due to inconsistent findings. Studies in liver tissue report reduced PE content in patients with MASH, while serum PE levels have been observed to increase in MASLD patients [22,23]. Interestingly, studies in liver cell models show that increased PE levels impair mitochondrial function, promote lipid accumulation, and increase fibrosis [23]. Our current study detected lower PE content in HDLs, aligning with liver findings but contradicting serum results. Additional research is essential to determine whether PE actively drives MASLD progression or whether the observed changes reflect compensatory changes in lipid metabolism.

HDL-LPC is primarily generated from PC by the action of Lecithin: cholesterol acyltransferase (LCAT). Moreover, LPC hepatic secretion and PC breakdown by lipoprotein-phospholipase A2 (Lp-PLA2) are also considered responsible for total plasma LPC levels [24]. Although the findings regarding serum LPC concentration in MASLD patients are inconsistent [22], the analysis of the liver lipidome in patients with MASH revealed an increase in LPC [14,16]. The latter was correlated with disease severity, suggesting that LPC may be involved in lipotoxicity and in the transition from simple steatosis to MASH. In our study, the content of HDLs-LPC was found significantly higher in MASLD patients compared to controls and comparable between MASLD and CAD patients. This finding could be attributed to increased hepatic secretion or enhanced activity of LCAT and Lp-PLA2 enzymes. Of note, the activity of LCAT was reported to be elevated in MASLD, as inferred from a Fatty Liver Index > 60 [25], and Lp-PLA2 levels were found increased in MASLD patients [26].

The percentage of total sphingolipids in HDLs was higher in MASLD patients compared to both controls and CAD patients, mainly due to the higher content of the rest of the SLs attributed to ceramides. As far as ceramides content is concerned, little is known about their role in the structural and functional properties of HDL lipoproteins. This bioactive lipid represents only a minor fraction of total HDL lipid mass, and up to now, no previous studies on the HDL lipidome reported changes in ceramide content in these patients.

Ceramides play a pivotal role in the development and progression of MASLD. Studies demonstrated that ceramides levels were elevated not only in the hepatocytes but also in the plasma of MASLD patients [3,27]. Thus, the increase in the rest of the SLs attributed to ceramides content in HDLs observed in MASLD patients possibly reflects increased ceramide biosynthesis. Chronic inflammation and oxidative stress that are involved in the pathophysiology of MASLD both increase de novo ceramide synthesis [28]. Furthermore, ceramide itself, through the positive feedback mechanism, promotes cytokine secretion, resulting in further activation of pro-inflammatory pathways. Similarly, ceramides are involved in oxidative stress, induce mitochondria dysfunction, and promote cellular apoptosis [28].

Phospholipids also play a determinant role in the functionality of HDLs, modulating their surface charge and fluidity, and regulating the activity of the associated enzymes [29]. Phospholipid content has also a major impact on HDL size [30]. Depletion in phospholipids decreases particles’ size and reduces the conformational stability of apoAI, resulting in its dissociation from HDL [30].

SR-B1-mediated cholesterol efflux is strongly determined by the HDL phospholipid composition, specifically, PC and SM [31]. Rached et al. demonstrated that enrichment of HDLs in LPC is associated with impaired cholesterol efflux and compromised HDL activities [32]. Fadaei R et al. [33] demonstrated that HDL cholesterol efflux capacity is impaired in MASLD patients and is associated with subclinical atherosclerosis. In our study, the low HDL-PC and high HDL-LPC content observed in MASLD patients may contribute to impaired HDL cholesterol efflux capacity. Yancey et al. demonstrated that enrichment of a particle with PC increases its ability to remove cholesterol from peripheral tissues [31]. Moreover, PC is also actively involved in the anti-inflammatory actions of HDL by inhibiting the expression of endothelial pro-inflammatory molecules [34]. Thus, the low percentage of HDL-PC observed in MASLD patients could reduce or even inhibit the SR-B1-dependent influx and lead to impaired cardioprotective properties of HDL. Finally, SR-B1 has a higher affinity for negatively charged, PI-enriched HDL particles, so reduced PI in MASLD may hinder cholesterol clearance [35].

The fatty acid pattern of the HDL lipids was altered gradually from controls to MASLD and then to CAD patients. SFA and MUFA were gradually increased from controls to MASLD and then to CAD patients, whereas UFA and PUFA (total and individual) were gradually decreased in the same direction.

An increase in SFA content has been reported in the hepatocytes and serum of MASLD patients [14,36]. SFAs induce lipotoxicity in fatty liver by activating receptors of cellular apoptosis and have been correlated with disease severity [37]. Also, their role in CVD risk has been well documented. SFA are associated with increased CVD risk and impaired endothelial function [38,39]. Specifically, SFAs promote pro-inflammatory responses and induce endothelial injury by impeding endothelial repair. Clinical studies have shown that SFAs impair the anti-inflammatory properties of HDLs, whereas PUFAs improve substantially the endothelial function [40].

In MASLD patients, HDLs were depleted in individual PUFAs compared to the control group. These findings are consistent with lipidomic studies on the liver and plasma, and notably, align with the previous study on HDL composition in patients with MASLD [10,14,22]. However, in this recent analysis of HDL composition, PUFA-PL and PUFA-FFA were found to be reduced, whereas we report decreased content in whole PUFA of HDL particles in patients with MASLD [10]. Although the levels of n-3 and n-6 PUFA are largely affected by diet, and poor nutritional intake may be the reason for our findings, evidence from lipidomic studies suggest that their biosynthetic pathways are also impaired. The multistep process of PUFA synthesis involves several elongases and desaturases. In fact, the activities of fatty acid desaturase 1 (FADS1) and elongase 6 (ELOVL6) were decreased in MASH patients, and their role in the progression of simple steatosis to MASH is a subject currently under intense investigation [36]. Eicosapentaenoic and docosahexaenoic acid have beneficial effects for HDL functionality and seem to decrease the cardiovascular risk [39].

Enrichment of HDLs in TG and depletion in CE are the most common abnormalities of the HDL lipidome in cardiometabolic diseases [41,42,43] and in CAD [38,39]. Compared to controls, these changes failed to reach statistical significance, potentially because the majority of MASLD patients were newly diagnosed. However, compared to CAD, MASLD patients presented with significantly higher TG and lower CE content in HDLs.

This lipid modification is attributed to the enhanced heteroexchange of TG and CE between VLDL and HDL [29], a process that is mediated by CE transfer protein. CETP activity has been found to be increased in patients with MASLD [33]. Mechanistically, the increased flux of FFA from adipose tissue, the activation of de novo lipogenetics pathways, and the overproduction of TG-rich VLDL lipoproteins, in the setting of MASLD, account for the enhanced activity of CETP and the increased ratio of TG to CE in HDL [44]. In our study, compared to controls, the increase in TGs and the reduction of CE content was not statistically significant, possibly because of the special features of MASLD patients suggested above. Nevertheless, even minor changes in the TG/CE ratio could affect HDL properties and its atheroprotective functions. Compensatory mechanisms in early MASLD may explain our findings, highlighting the need for further studies on MASH and advanced fibrosis.

These alterations in particles’ core influence the physical properties, stability, and structural integrity of HDLs [45]. Enrichment in TG increases the fluidity of the hydrophobic core, resulting in unstable HDLs with increased catabolism [46]. Particularly, hepatic lipase, an important lipolytic enzyme involved in HDL remodeling, metabolizes these TG-enriched particles, accelerating their clearance [46]. Moreover, increased HDL-TG content affects the conformation of the central and C-terminal domains of apoAI, resulting in the detachment of apoAI and enhanced clearance of HDL from circulation [45]. Changes in TG and CE content impair reverse cholesterol transport by reducing LCAT activity, which is crucial for cholesterol esterification [47]. Additionally, TG-enriched, CE-depleted HDLs are inefficient in donating CE via SR-B1, compromising a key cholesterol removal pathway and impairing HDL’s protective role [48]. These mechanistic insights highlight the importance of detailed HDL lipid composition analysis in understanding cardiovascular risk. In this context, familial hypercholesterolemia (FH)—a model disorder of lipid metabolism with well-defined clinical features—may particularly benefit from advanced lipidomic profiling, which could uncover novel molecular alterations and enhance risk stratification [49].

This study has limitations that should be acknowledged. Its cross-sectional design limits the ability to infer causality. No data are available on insulin resistance and inflammation biomarkers, as well as diet.

## 4. Materials and Methods

### 4.1. Participants

Fifty-one patients with a recently established diagnosis of MASLD were recruited from the Hepatology outpatient clinic of University Hospital of Ioannina. The diagnosis of MASLD was established with liver ultrasonography evidence of fatty liver and the presence of at least one cardiometabolic risk factor according to criteria proposed by the European Association for the Study of the Liver [2]. The cardiometabolic risk factors considered were (i) body mass index (BMI) ≥ 23 kg/m^2^ or waist circumference ≥ 90 cm (male) and ≥85 cm for (female); (ii) fasting serum glucose ≥ 100 mg/dL, type 2 diabetes, or treatment for type 2 diabetes; (iii) blood pressure ≥ 130/85 mmHg or antihypertensive medication; (iv) triglycerides ≥ 150 mg/dL or lipid-lowering treatment; and (v) HDL cholesterol ≤ 40 mg/dL for male and ≤50 mg/dL for female or lipid-lowering treatment. Individuals with a history of daily alcohol intake > 20 g for women and >30 g for men were excluded. Subjects with evidence of other causes of liver disease were also excluded by screening for viral hepatitis (hepatitis B and hepatitis C), for autoimmune liver disease (autoimmune hepatitis, sclerosing cholangitis, primary biliary cirrhosis), for Wilson’s disease, for hemochromatosis, and for alpha-1 antitrypsin deficiency. None of the subjects included were on medications known to cause hepatic steatosis.

A total of 40 patients with a confirmed diagnosis of acute coronary syndrome, without persistent elevation of the ST segment in the electrocardiograph (NSTE-ACS) and angiographically demonstrated three-(severe) vessel disease (defined as the presence of ≥50% diameter luminal narrowing in 3 of the major epicardial vessel systems) were admitted to the Coronary Care Unit of the University Hospital of Ioannina and participated in the study. All patients underwent diagnostic coronary angiography within 7−9 days after the onset of the symptoms. The diagnosis of NSTE-ACS was based on the criteria proposed by the European Society of Cardiology [50]. Patients who did not meet the criteria for NSTE-ACS after the initial evaluation were excluded from the study. Patients with a family history of premature cardiovascular disease, chronic renal disease, hepatic function impairment, chronic obstructive pulmonary disease, overt hyper-/hypothyroidism, or rheumatic diseases; patients on lipid-lowering drugs such as statins; and patients on blood pressure-lowering drugs that affect lipid metabolism (such as diuretics or beta blockers) were excluded from the study. All groups were matched for age, gender, and serum lipid profile (total, LDL, non-HDL, and HDL cholesterol; triglycerides), apoAI, and apoB to minimize the confounding effect of these parameters on the data analysis. The control group comprised 50 healthy individuals with no medical history.

This study was conducted as an observational cross-sectional investigation with prospective data collection. Serum samples and clinical data were obtained at the time of patient enrollment, specifically for the purposes of this research. Data collection and samples from all participants were conducted in accordance with the guidelines of the Scientific Committee of the University Hospital of Ioannina. Written consent was obtained from each participant before the study procedure was performed, and the study protocol was approved by the Ethics committee of the University Hospital of Ioannina.

### 4.2. Sample Collection and Preparation

After an overnight fast, venous blood samples were obtained from all study participants and from CAD patients within the first 12 h from the onset of angina symptoms to avoid lipoprotein changes. After centrifugation at 1500× *g* for 15 min, serum was separated for determination of biochemical parameters, and one 1,5 mL aliquot was stored until NMR analysis at −80 °C.

### 4.3. Biochemical Parameters

Total cholesterol and triglycerides were determined enzymatically and HDL cholesterol by a direct assay on an Olympus AU5400 Clinical Chemistry analyzer (Beckman, Hamburg, Germany). LDL cholesterol was calculated by the Friedewald formula, and non-HDL cholesterol was calculated as the difference between total cholesterol and HDL cholesterol.

### 4.4. Isolation and Lipid Extraction of HDL Lipoproteins

HDL lipoprotein particles were isolated from non-HDL lipoproteins by selective precipitation of the non-HDLs (apoB containing lipoproteins). A total of 1.5 mL of serum was mixed with 150 µL of a solution containing 10 g/L Dextran Sulfate (50 kDa) and 500 mmol/L MgCl_2_ (polyanions and divalent cations). HDL lipoproteins were collected from the supernatant, and their lipid content was extracted with methanol/chloroform (2:1) according to a modification of the Bligh and Dyer method [51], dried in a stream of nitrogen, and stored at −80 °C to avoid oxidative degradation [52]. The samples were redissolved in a 500 μL deuterated mixture of methanol/chloroform (2:1) and bubbled with nitrogen in order to remove oxygen just prior to recording the NMR spectrum.

#### H NMR Spectroscopy

The extracted HDL lipids were dissolved in 500 μL of deuterated methanol/chloroform (2:1, *v*/*v*). All NMR spectra were recorded on a 500 MHz Bruker Avance DRX NMR spectrometer (Bruker, Bremen, Germany) (NMR Center, University of Ioannina) operating at a field strength of 11.74 Tesla. A “zgpr” Bruker pulse program was applied, with the parameters as follows: 64 scans, 64 K data points with a 5000 Hz spectral width, and a 90° pulse. All free induction decays (FIDs) were multiplied by an exponential weighting function corresponding to the 0.3 Hz line-broadening factor prior to Fourier transformation. NMR spectra were phase- and baseline-corrected and referenced to the methanol signal (δ = 3.30 ppm) using TopSpin 4.4 software (Bruker Biospin Ltd., GmbH, Rheinstetten, Germany).

Quantification of the HDL lipids was based on the integration of selected signals in the proton NMR spectrum (Table S1), corrected for the number of protons and then normalized with respect to the signal from the cholesterol C18 methyl group at 0.68 ppm. The lipid composition of HDL lipoproteins was expressed as percentages of the total lipids of HDL lipoprotein particles. Spectral assignments and quantification of HDL lipid peaks were described previously [52].

### 4.5. Statistical Analysis

Univariate analysis: All data were expressed as the mean value ± SD. Group comparisons were performed using one-way analysis of variance (ANOVA), followed by a least significant differences (LSD) test for pairwise comparisons. A *p*-value < 0.05 was considered to indicate statistical significance.

Multivariate analysis: For the untargeted analysis, HDL lipids data were normalized using MetaboAnalyst v.6.0, and an unsupervised principal component analysis (PCA) was applied to detect clustering trends between groups and the presence of potential outliers. Then, a supervised partial least squares discriminant analysis (PLS-DA) was carried out to maximize the separation between the groups according to their different HDL lipid profiles. The performance of the models was tested by a 10-fold cross-validation, where R2 and Q2 parameters indicate the proportion of data variance and the predictive ability of the model, respectively. Permutation tests were also carried out to check overfitting and the validation of the resulting PLS-DA models. The PLS-DA score plot, where each point represents a sample, was used to visualize any grouping trend or separation, whereas the variable importance in projection (VIP) plot was used to highlight the most important HDL lipid components responsible for the grouping trend or separation seen in the PLS-DA model.

## 5. Conclusions

In summary, we report that the HDL lipid composition, and especially the phospholipid pattern and fatty acid composition, is altered in patients with MASLD. Interestingly, these alterations have been correlated mostly with the lipid changes observed in hepatocytes of MASLD patients. The reduced PC and PI content and increased LPC content of HDL particles in MASLD patients may impair HDL functionality. Moreover, the gradual shift in fatty acid composition from controls to MASLD and then to CAD patients suggests that MASLD may contribute to cardiovascular disease progression by altering lipid metabolism and compromising HDL function.

These findings provide new insights into HDL remodeling in MASLD and its potential role in cardiovascular risk. Although further validation in larger and prospective cohorts is required, HDL lipidomic profiling may eventually serve as a complementary tool for cardiovascular risk stratification. Such stratification could help identify individuals who may benefit from more intensive preventive strategies, including tighter control of metabolic risk factors and earlier cardiometabolic monitoring. Furthermore, characterizing specific HDL lipid alterations could inform the development of targeted treatments aimed at restoring HDL functionality—such as therapies that modulate phospholipid content or fatty acid composition—to reduce cardiovascular risk in this high-risk population.

## Figures and Tables

**Table 1:** Demographic data and serum lipid parameters of the study participants.

**Total** **(n = 141)**	**Controls** **(n = 50)**	**MASLD ^1^** **(n = 51)**	**CAD ^2^** **(n = 40)**	***p*** **-Value**
Age (years)	54.3 ± 12.4	51.4 ± 12.9	55.2 ± 8.5	NS
Gender (Males/Females)	26/24	23/28	22/18	
**Serum Lipid Parameters (mg/dL)**				
Total Cholesterol	197.9 ± 30.5	207.6 ± 47.7	204.3 ± 45.4	NS
Triglycerides	121.9 ± 33.7	132.4 ± 48.6	131.4 ± 64.8	NS
HDL Cholesterol	48.9 ± 11.5	51.3 ± 12.4	48.4 ± 10.1	NS
LDL Cholesterol	124.7 ± 29.4	130.0 ± 39.9	129.5 ± 43.2	NS
non-HDL Cholesterol	149.0 ± 28.4	156.2 ± 43.5	155.8 ± 42.3	NS

^1^ MASLD: metabolic dysfunction-associated steatotic liver disease; ^2^ CAD: coronary artery disease. NS: Statistically not significant.

**Table 2:** HDL composition of the major lipid classes expressed as mols/100 mols of total lipids.

**Total** **(n = 141)**	**Controls** **(n = 50)**	**MASLD ^1^** **(n = 51)**	**CAD ^2^** **(n = 40)**
**% Cholesterol, total**	41.87 ± 1.54	42.13 ± 1.79	40.63 ± 1.87 **^,+++^
Esterified (EC)	32.12 ± 1.37	31.98 ± 1.29	30.89 ± 1.69 ***^,+++^
Free (FC)	9.75 ± 0.84	10.15 ± 0.96 *	9.74 ± 0.86 ^+^
**% Triglycerides (TG)**	4.65 ± 1.12	4.74 ± 1.72	5.93 ± 1.11 ***^,+++^
**% Phospholipids (PLs), total**	53.47 ± 1.66	53.14 ± 2.06	53.44 ± 1.83
% Core Lipids, total	36.77 ± 1.75	36.72 ± 1.82	36.82 ± 1.85
% Surface Lipids, total	63.23 ± 1.75	63.28 ± 1.82	63.18 ± 1.85
FC/PLs	0.18 ± 0.02	0.19 ± 0.02 *	0.18 ± 0.02 ^+^
EC/FC	3.32 ± 0.34	3.18 ± 0.30 *	3.20 ± 0.33
EC/Total Cholesterol	0.77 ± 0.02	0.76 ± 0.02 *	0.76 ± 0.02
TG/EC	0.14 ± 0.04	0.15 ± 0.06	0.19 ± 0.04 ***^,+++^

^1^ MASLD: metabolic dysfunction-associated steatotic liver disease; ^2^ CAD: coronary artery disease. * *p* < 0.05, ** *p* < 0.01, and *** *p* < 0.001 compared to controls; ^+^ *p* < 0.05 and ^+++^ *p* < 0.001 compared to patients with MASLD.

**Table 3:** Phospholipid profiling of HDL lipoproteins of the three groups studied expressed as mols/100 mols of total lipids.

**Total** **(n = 141)**	**Controls** **(n = 50)**	**MASLD ^1^** **(n = 51)**	**CAD ^2^** **(n = 40)**
**Glycerophospholipids (GPLs), total**	41.37 ± 1.55	39.98 ± 2.22 **	41.99 ± 2.14 ^+++^
Phosphatidylcholine (PC)	34.22 ± 1.35	33.13 ± 2.77 *	33.41 ± 2.17
Lysophosphatidylcholine (LPC)	2.36 ± 0.40	2.69 ± 0.56 **	2.70 ± 0.53 **
Phosphatidylethanolamine (PE)	1.24 ± 0.29	0.94 ± 0.26 ***	0.99 ± 0.28 ***
Phosphatidylinositol (PI)	2.13 ± 0.58	1.47 ± 0.43 ***	2.50 ± 1.21 *^,+++^
Rest of the GPLs ^a^	1.59 ± 1.27	1.78 ± 1.76	2.40 ± 1.74 *
**Ether Glycerolipids (Ether GLs), total**	4.99 ± 0.84	5.34 ± 1.03	4.39 ± 0.94 **^,+++^
Plasmalogens	1.77 ± 0.30	1.71 ± 0.37	1.88 ± 0.48 ^+^
Rest of the ether GLs ^b^	3.22 ± 0.82	3.63 ± 0.96 *	2.51 ± 0.83 ***^,+++^
**Sphingolipids (SLs), total**	7.11 ± 0.69	7.82 ± 0.71 ***	7.06 ± 0.89 ***^,+++^
Sphingomyelin (SM)	6.19 ± 0.97	6.34 ± 1.01	5.91 ± 0.89 ^+^
Rest of the SLs ^c^	0.92 ± 0.72	1.48 ± 0.78 ***	1.15 ± 0.59 ^+^
PC/SM	4.61 ± 0.50	5.01 ± 0.78 **	4.56 ± 0.55 ^++^
PC/LPC	14.87 ± 2.45	12.84 ± 2.76 ***	12.87 ± 2.75 **
SM/LPC	2.67 ± 0.54	2.49 ± 0.84	2.23 ± 0.39 **

^1^ MASLD: metabolic dysfunction-associated steatotic liver disease; ^2^ CAD: coronary artery disease. * *p* < 0.05, ** *p* < 0.01, and *** *p* < 0.001 compared to controls; ^+^ *p* < 0.05, ^++^ *p* < 0.01, and ^+++^ *p* < 0.001 compared to patients with MASLD. a: mainly phosphatidylserine, phosphatidylglycerol; b: mainly PAF; c: mainly ceramide.

**Table 4:** Fatty acid profile of HDL lipoproteins expressed as mol/100 mols of total fatty acids.

**Total** **(n = 141)**	**Controls** **(n = 50)**	**MASLD ^1^** **(n = 51)**	**CAD ^2^** **(n = 40)**
**SFA**	39.42 ± 3.37	41.07 ± 3.30 *	45.18 ± 5.11 ***^,+++^
**UFA**	60.58 ± 3.37	58.93 ± 3.30 *	54.82 ± 5.11 ***^,+++^
**MUFA**	7.98 ± 2.17	8.75 ± 3.04	9.58 ± 2.26 **
**PUFA**	52.60 ± 3.12	50.18 ± 3.29 **	45.24 ± 5.41 ***^,+++^
LA	19.57 ± 1.76	16.72 ± 2.07 ***	15.69 ± 2.17 ***^,+^
EPA + AA	12.28 ± 1.39	9.85 ± 1.68 ***	9.14 ± 0.88 ***^,+^
DHA	4.09 ± 0.76	3.33 ± 0.61 ***	2.86 ± 0.47 ***^,+++^
**SFA/UFA**	0.66 ± 0.10	0.70 ± 0.10	0.84 ± 0.18 ***^,+++^
**SFA/PUFA**	0.76 ± 0.11	0.82 ± 0.11 *	1.03 ± 0.25 ***^,+++^

^1^ MASLD: metabolic dysfunction-associated steatotic liver disease; ^2^ CAD: coronary artery disease. * *p* < 0.05, ** *p* < 0.01, and *** *p* < 0.001 compared to controls; ^+^ *p* < 0.05 and ^+++^ *p* < 0.001 compared to patients with MASLD.

## Data Availability

Data are contained within the article.

## Supplementary Materials

The following supporting information can be downloaded at: https://www.mdpi.com/article/10.3390/ijms26136148/s1.

## Abbreviations

The following abbreviations are used in this manuscript:

^1^H NMR	proton nuclear magnetic resonance
ABCA1	ATP-binding cassette subfamily A member 1
apoAI	apolipoprotein-AI
CE	cholesteryl ester
Cer	ceramide
CETP	cholesteryl ester transfer protein
CVD	cardiovascular disease
HDL	high-density lipoprotein
LCAT	lecithin-cholesterol acyltransferase
LDL	low-density lipoprotein
LPC	lysophosphatidylcholine
LpPLA2	lipoprotein phospholipase A2
MASLD	metabolic dysfunction-associated steatotic liver disease
MASH	metabolic dysfunction-associated steatohepatitis
PC	phosphatidylcholine
PE	phosphatidylethanolamine
PI	phosphatidylinositol
PUFA	polyunsaturated fatty acids
SFA	saturated fatty acids
SM	sphingomyelin
SR-B1	scavenger receptor class B type 1
SREBPs	sterol regulatory element-binding proteins
TG	triglycerides
VLDL	very-low-density lipoprotein
