# Supplementary material for: Altered HDL Phospholipid and Fatty Acid Profile in MASLD: A Possible Explanation for the Increased CVD Risk
Source: Int J Mol Sci. 2025 Jun 26;26(13):6148. doi: 10.3390/ijms26136148 (PMC12250491; doi:10.3390/ijms26136148)
Supplement: Supplementary file 1 [file ijms-26-06148-s001.zip › ijms-3661155-supplementary.pdf]

Supplementary Materials

Altered HDL Phospholipid and Fatty Acid Profile in MASLD: A Possible Explanation for the Increased CVD Risk

Sofia Kartsoli [1], Christina E. Kostara [2], Athanasios Papathanasiou [3], Vasilis Tsimihodimos [3], Eleni T. Bairaktari [2] and Dimitrios K. Christodoulou [1,*]

[1] Department of Gastroenterology, School of Health Sciences, Faculty of Medicine, University of Ioannina, 45110 Ioannina, Greece
[2] Laboratory of Clinical Chemistry, School of Health Sciences, Faculty of Medicine, University of Ioannina, 45110 Ioannina, Greece
[3] Department of Internal Medicine, School of Health Sciences, Faculty of Medicine, University of Ioannina, 45110 Ioannina, Greece
* Correspondence: dchristo@uoi.gr

Supplementary Data: Figure S1 shows a representative ^{1}H NMR spectrum of HDL lipid extract from a healthy individual that contains signals attributed to protons of cholesterol, in free and esterified form, the headgroup and backbones of phospholipids (glycerophospholipids and sphingolipids), the glycerol backbone and esterified fatty acids.

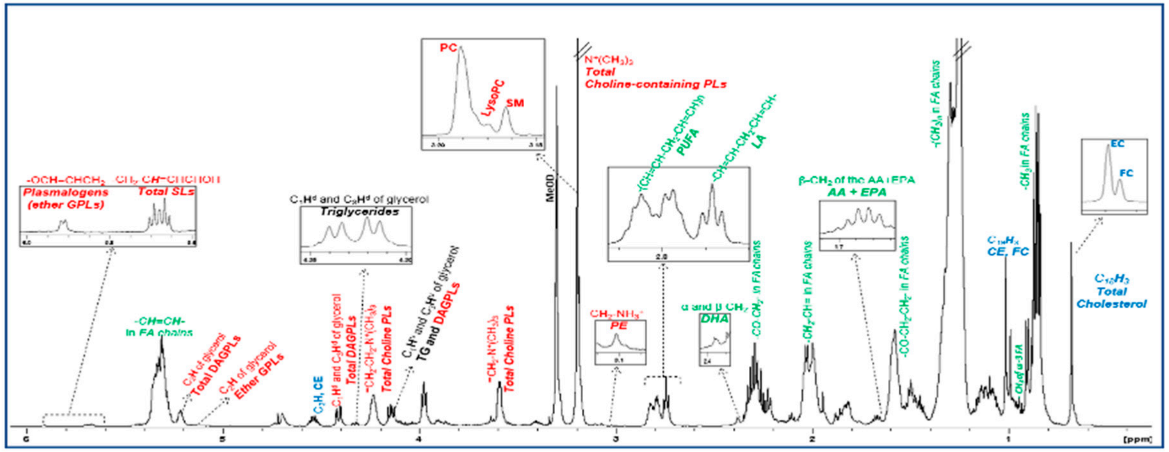

Figure S1. ^{1}H NMR spectrum of an HDL lipid extract from healthy individual. Signals attributed to Phospholipids (colored in red), Triglycerides (colored in black), Fatty Acids (colored in green) and to Cholesterol (colored in blue). Peak assignments are summarized in Table S1.

Key: AA, arachidonic acid; CE, cholesteryl ester; DAGPLs, Diacyl glycerophospholipids; DHA, docosahexaenoic acid; EPA, eicosapentaenoic acid; FA, fatty acids; FC, free cholesterol; GPLs, glycerophospholipids; LA, Linoleic acid; LPC, lysophosphatidylcholine; PC, phosphatidylcholine; PE, phosphatidylethanolamine; PUFA, polyunsaturated fatty acids; SLs, Sphingolipids; SM, sphingomyelin; TG, triglycerides; UFA, Unsaturated fatty acids.

Table S1. Protons and Chemical Shift (in ppm) for the lipid constituents and headgroups identified in HDL lipid extract by NMR and selected signals for Lipid Quantification

**Lipid constituents and headgroups**	**^{1}H NMR signal assignment**	**Chemical Shift (ppm)**	**Quantification of lipids from selected well-resolved NMR signals**
**Cholesterol molecule**	$\mathbf{C_{18}H_3}$	0.68	**Total Cholesterol, FC, CE**
	$\mathbf{C_{26}H_3, C_{27}H_3, C_{21}H}$	0.87	
	$\mathbf{C_{19}H_3}$	1.00	
	$\mathbf{C_3H}$	3.40	
	$\mathbf{C_6H}$	5.36	
**Glycerol backbone**	$C_1\mathbf{H}^u$ and C_3H^u of glycerol backbone of TG and DAGPLs	4.16	
	$C_1\mathbf{H}^d$ and C_3H^d of glycerol backbone of TG	4.32	**TG**
	$C_1\mathbf{H}^d$ and C_3H^d of glycerol backbone of DAGPLs	4.40	
	$C_2\mathbf{H}$ of glycerol backbone in ether glycerophospholipids	5.15	**Ether GPLs**
	$C_2\mathbf{H}$ of glycerol backbone in total DAGPLs	5.18	**Total Diacyl glycerophospholipids (DAGPLs)**
	$C_2\mathbf{H}$ of glycerol backbone in TG	5.22	
**Sphingosine moiety**	$-CH_2-CH=C\mathbf{H}CHOH$	5.40	
	$-CH_2-C\mathbf{H}=CHCHOH$	5.70	**Total SLs**
**Head-group and substituent**	$-CH_2-CH_2-N^+(C\mathbf{H}_3)_3$ (choline)	3.20	**Total choline-containing PLs (PC, SM, LPC)**
	$-CH_2-C\mathbf{H}_2-N^+(CH_3)_3$	3.59	
	$-C\mathbf{H}_2-CH_2-N^+(CH_3)_3$	4.24	
	$-CH_2-C\mathbf{H}_2-NH_3^+$ (ethanolamine)	3.10	**PE**
	$-OC\mathbf{H}=CHCH_2$	5.90	**PLA (ether GPLs)**
**Fatty acid chains**	ω-$C\mathbf{H}_3$ (methyl) in fatty acyl chains	0.88	
	ω-$C\mathbf{H}_3$ (methyl) of total omega-3 FA	0.95	
	$-(C\mathbf{H}_2)_n-$ (methylene) in fatty acyl chains	1.30	
	$-CO-CH_2-C\mathbf{H}_2-$ (β-methylene) in the fatty acyl chains	1.59	
	β-$C\mathbf{H}_2$ (β-methylene) of the sum of AA+EPA	1.67	**AA (20:4 ω-6) + EPA (20:5 ω-3)**
	$-C\mathbf{H}_2-CH=$ (allylic) in fatty acyl chains	2.04	**UFA**

	-CO-C**H**$_2$ (α-methylene) in the fatty acyl chains	2.30	**Total FA**
	α and β C**H**$_2$ (methylene) of DHA	2.38	**DHA (22:6 ω-3)**
	-CH=CH-C**H**$_2$-CH=CH- of linoleic acid	2.75	**LA (18:2 ω-6)**
	-(CH=CH-C**H**$_2$-CH=CH)n, n > 1 in the fatty acyl chains	2.80	**PUFA**
	-C**H**=C**H**- in the fatty acyl chains	5.36	

Key AA, arachidonic acid; **CE**, cholesteryl ester; **DAGPLs**, Diacyl glycerophospholipids; **DHA**, docosahexaenoic acid; **EPA**, eicosapentaenoic acid; **FA**, fatty acids; **FC**, free cholesterol; **GPLs**, glycerophospholipids; **LA**, Linoleic acid; **LPC**, lysophosphatidylcholine; **PC**, phosphatidylcholine; **PE**, phosphatidylethanolamine; **PLA**, plasmalogens; **PUFA**, polyunsaturated fatty acids; **SLs**, Sphingolipids; **SM**, sphingomyelin; **TG**, triglycerides; **UFA**, Unsaturated fatty acids.
